# Impact of combined FDG-PET/CT and MRI on the detection of local recurrence and nodal metastases in thyroid cancer

**DOI:** 10.1186/s40644-016-0096-y

**Published:** 2016-11-03

**Authors:** Johann-Martin Hempel, Roman Kloeckner, Sandra Krick, Daniel Pinto dos Santos, Simin Schadmand-Fischer, Patrick Boeßert, Sotirios Bisdas, Matthias M. Weber, Christian Fottner, Thomas J. Musholt, Mathias Schreckenberger, Matthias Miederer

**Affiliations:** 1 Department of Radiology, Diagnostic and Interventional Neuroradiology, Eberhard Karls University Tübingen, Hoppe-Seyler-Str. 3, D-72076 Tübingen, Germany; 2 Department of Diagnostic and Interventional Radiology, Johannes Gutenberg-University Medical Center, Langenbeckstr. 1, D-55131 Mainz, Germany; 3 Department of Nuclear Medicine, Johannes Gutenberg-University Medical Center, Langenbeckstr. 1, D-55131 Mainz, Germany; 4 Department of Otolaryngology and Head and Neck Surgery, AMEOS Hospital Haldensleben, Kiefholzstr. 4 & 27, D-39340 Haldensleben, Germany; 5 Department of Endocrinology and Metabolism, I. Medical Clinic, Johannes Gutenberg-University Medical Center, Langenbeckstr. 1, D-55131 Mainz, Germany; 6 Clinic of General, Visceral- and Transplantation Surgery, Endocrine Surgery Section, Johannes Gutenberg-University Medical Center, Langenbeckstr. 1, D-55131 Mainz, Germany; 7 Department of Neuroradiology, National Hospital of Neurology and Neurosurgery, University College London Hospitals, London, United Kingdom

**Keywords:** PET/CT, MRI, Recurrent thyroid cancer, Recurrent thyroid carcinoma, Thyroid cancer, Nodal metastases, Lymph node metastases, Consensus reading, Iodine-negative, Negative ultrasound

## Abstract

**Background:**

Suspected recurrence of thyroid carcinoma is a diagnostic challenge when findings of both a radio iodine whole body scan and ultrasound are negative. PET/CT and MRI have shown to be feasible for detection of recurrent disease. However, the added value of a consensus reading by the radiologist and the nuclear medicine physician, which has been deemed to be helpful in clinical routines, has not been investigated. This study aimed to investigate the impact of combined FDG-PET/ldCT and MRI on detection of locally recurrent TC and nodal metastases in high-risk patients with special focus on the value of the multidisciplinary consensus reading.

**Materials and methods:**

Forty-six patients with suspected locally recurrent thyroid cancer or nodal metastases after thyroidectomy and radio-iodine therapy were retrospectively selected for analysis. Inclusion criteria comprised elevated thyroglobulin blood levels, a negative ultrasound, negative iodine whole body scan, as well as combined FDG-PET/ldCT and MRI examinations.

Neck compartments in FDG-PET/ldCT and MRI examinations were independently analyzed by two blinded observers for local recurrence and nodal metastases of thyroid cancer. Consecutively, the scans were read in consensus. To explore a possible synergistic effect, FDG-PET/ldCT and MRI results were combined. Histopathology or long-term follow-up served as a gold standard.

For method comparison, sensitivity, specificity, positive and negative predictive values, and diagnostic accuracy were calculated.

**Results:**

FDG-PET/ldCT was substantially more sensitive and more specific than MRI in detection of both local recurrence and nodal metastases. Inter-observer agreement was substantial both for local recurrence (κ = 0.71) and nodal metastasis (κ = 0.63) detection in FDG-PET/ldCT. For MRI, inter-observer agreement was substantial for local recurrence (κ = 0.69) and moderate for nodal metastasis (κ = 0.55) detection. In contrast, FDG-PET/ldCT and MRI showed only slight agreement (κ = 0.21). However, both imaging modalities identified different true positive results. Thus, the combination created a synergistic effect. The multidisciplinary consensus reading further increased sensitivity, specificity, and diagnostic accuracy.

**Conclusions:**

FDG-PET/ldCT and MRI are complementary imaging modalities and should be combined to improve detection of local recurrence and nodal metastases of thyroid cancer in high-risk patients. The multidisciplinary consensus reading is a key element in the diagnostic approach.

**Electronic supplementary material:**

The online version of this article (doi:10.1186/s40644-016-0096-y) contains supplementary material, which is available to authorized users.

## Background

Thyroid carcinoma (TC) is a rare malignancy. Nonetheless, the incidence has grown markedly since the early 1990s. In 2010 the standardized incidence in the US was estimated at 6.0/100,000 in males and 17.3/100,000 in females [1, 2]. In Europe, the standardized incidence ranges from 2.03/100,000 to 5.0/100,000 in males and from 5.65/100,000 to 15.50/100,000 in females [3]. TC has a relatively high rate of local recurrence and lymph node or soft tissue metastases; estimates range between 20 and 30 % [4, 5]. Distant metastases develop in up to 15 % of cases, primarily as pulmonary metastases, followed by bone metastases [6]. Prognosis primarily depends on the site of recurrence and the subsequent treatment, and early diagnosis of recurrence is of utmost importance to determine whether or not a salvage surgery is possible. Therefore, a close postoperative follow-up regime is mandatory [7].

Currently, the most frequently used modalities for post-thyroidectomy follow-up examinations are neck ultrasound (US), measuring thyroglobulin (Tg) blood levels, and radioiodine ^131^I-whole-body scan (I-WBS) [7–9]. However, 20–40 % of the patients with recurrent TC or nodal metastases lose their ability to accumulate radioactive iodine due to tumor cell dedifferentiation. Thus, they are not visible on I-WBS. In this case, alternative imaging modalities are needed, such as ^18^F-fluorodeoxyglucose Positron emission tomography (FDG-PET), ^18^F-fluorodeoxyglucose-PET/computed tomography (FDG-PET/CT), Magnetic resonance imagining (MRI), or even ^18^F-fluorodeoxyglucose-PET/MRI (FDG-PET/MRI) [5, 10–12].

FDG-PET was shown to be a useful tool in the localization of disease in patients with elevated Tg levels and negative US and I-WBS [13, 14]. It was quickly outperformed by FDG-PET/CT, which combined the advantages of metabolic and morphologic imaging and thus allowed for improved anatomic localization and correlation of focal metabolic activity. FDG-PET/CT is now commonly accepted as the method of choice for post-thyroidectomy patients with increased Tg blood levels and negative I-WBS [11, 12, 15–18]. For distant metastases, particularly of the lung and bones, FDG-PET/CT reaches both sensitivity and specificity up to 1.00 [14, 15, 19]. Nonetheless, the sensitivity and specificity of FDG-PET/CT for detecting locally recurrent or metastatic TC is still relatively low and ranges between 0.46 to 1.00 and 0.66 to 1.00, respectively [5, 12, 17]. Furthermore, recurrent, residual, or metastatic tissue does not always accumulate ^18^FDG, and thus remains undetectable by FDG-PET/CT. Hence, supplementary imaging modalities are occasionally required.

MRI is the primary imaging modality for soft tissue tumors due to its excellent soft tissue contrast, additional functional imaging capabilities, and reduced dental metal artifacts in the head and neck, as compared to FDG-PET/CT [20–23]. MRI of the neck is mainly used for planning the surgical approach and postoperative follow-up in head and neck cancer and TC patients. Its specific application for TC has frequently been described in the literature [22, 24, 25]. The sensitivity and specificity of MRI in detecting locally recurrent or metastatic TC ranges from 0.76 to 0.95 and 0.51 to 0.98, respectively [4, 22, 24, 26]. Because of its superior soft tissue contrast and its ability to detect non-iodine-avid and FDG-negative tumor tissue, MRI appears to be a powerful complementary imaging modality to FDG-PET/CT [4, 22, 23, 26, 27]. Additionally, gadolinium-based MRI-contrast media avoids iodine contamination before curative radioiodine therapy [4, 23]. PET/MRI is a new hybrid imaging modality that has been applied to TC. Initial results suggested PET/MRI was superior to PET/CT in detecting iodine-positive lesions [28], but for evaluating the primary disease in the neck area it performed as well as contrast-enhanced PET/CT [29]. However, PET/CT was superior to PET/MRI in assessing pulmonary status [30].

A systematic assessment of the added value of ^18^F-fluorodeoxyglucose-PET/low dose computed tomography (FDG-PET/ldCT) and MRI for detecting locally recurrent TC or cervical nodal metastases has not yet been reported in the literature. Also, the added value of a consensus reading between a nuclear medicine physician and a radiologist, which we have found to be very helpful in clinical routine, has not been investigated. Therefore, this study aimed to evaluate the impact of combined FDG-PET/ldCT and MRI on the detection of local recurrence and cervical nodal metastases of TC. Furthermore, it attempted to quantify the value of a multidisciplinary consensus reading.

## Methods

### Study design, ethics, and study group

This study followed all procedures laid out in the ethical standards of the responsible committee on human experimentation and with the revised version of the Helsinki Declaration of 1975. All patients were referred to departments of radiology and nuclear medicine as indicated by clinical need and received sequential MRI and FDG-PET/ldCT scans for postoperative follow-up. No additional radiation dose was applied. Patient records and information were de-identified prior to analysis by the department’s information technology service. Institutional review board approval was waived, given that this study utilized the retrospective analysis of blinded clinical data.

This was a retrospective diagnostic study. Forty-six consecutive TC patients who underwent thyroidectomy and radio iodine therapy with ongoing suspicion of recurrent local or metastatic disease were reviewed by our multidisciplinary tumor board between June 2008 and November 2012. The inclusion criteria included elevated Tg blood level, negative ultrasound and I-WBS (diagnostic rhTSH stimulated I-WBS with administered activity of 370 or 3700 MBq) as well as combined FDG-PET/ldCT and MRI examinations that occurred within 72 h of one another. Tg blood levels above 2 μg/l were defined as clearly pathological. However, in some patients with high risk profile (e.g., lymph node metastases in clinical history) Tg values below 2 μg/l were considered as suspicious. Patients with a medullary subtype of TC were excluded.

Table 1 shows demographic and disease-related information of the study patients.

**Table 1:** Descriptive demographic and disease-related data of all study patients

Total number of consecutive study patients		46
Age (median, interquartile range)		70 (58-80)
Sex	Male	27 (59 %)
	Female	19 (41 %)
Tg blood levels (median, interquartile range)	Under TSH suppression	5.35 μg/l (0,35-79,22)
	Under TSH stimulation	31.4 μg/l (3,47-454,75)
Histopathology	Follicular	16 (35 %)
	Papillary	25 (54 %)
	Poorly differentiated	4 (9 %)
	Anaplastic	1 (2 %)
Stage of disease	I	3 (6 %)
	II	2 (4 %)
	III	5 (11 %)
	Iva	8 (18 %)
	IVb	2 (4 %)
	IVc	24 (53 %)
	x	2 (4 %)
Primary tumor	Ia	2 (4 %)
	Ib	4 (9 %)
	2	8 (18 %)
	3	16 (35 %)
	4	12 (25 %)
	x	4 (9 %)
Regional lymph nodes	0	17 (37 %)
	1a	9 (20 %)
	1b	15 (33 %)
	x	5 (10 %)
Distant metastasis	0	16 (35 %)
	1	23 (50 %)
	x	7 (15 %)

Staging of thyroid carcinoma according to the 7^th^ edition of AJCC Cancer Staging Manual 2010

*TSH* thyroid stimulating hormone, *Tg* thyroglobulin

### Procedures and techniques

#### PACS-Viewer

Images were analyzed using the TeraRecon Aquarius thin client® viewer (TeraRecon, Inc., CA, USA).

#### FDG-PET/ldCT

PET/CT of the whole body was acquired on a Gemini® TF 16 PET/CT (Philips, Best, the Netherlands) 60 min after the application of ^18^F-fluorodeoxyglucose (2–2.5 MBq/kg). A fasting period of more than six hours before application was mandatory for all patients. For attenuation correction, low dose CT without application of contrast media was used.

#### MRI

Examinations of the cervical region were acquired with Magnetom Sonata® 1.5 T, Magnetom Avanto® 1.5 T, Magnetom Espree® 1.5 T, and Magnetom Skyra® 3 T (all Siemens Healthcare, Erlangen, Germany).

Due to variations between the scan protocols, only axial short tau inversion recovery (STIR)-weighted sequence, axial T1-weighted turbo spin echo (TSE) sequence, and Gadolinium contrast media enhanced (Dotarem®, Guerbet, France) axial T1-weighted TSE sequences with fat suppression were selected for study analysis.

#### Image analysis and definition of results

The FDG-PET/ldCT studies were independently analyzed by two blinded physicians who both had board certification in nuclear medicine and with 9 (MM) and 25(MS) years of professional experience. The MRI studies were independently analyzed by two blinded physicians who specialized in diagnostic radiology and who had 27 (SSF) and 5 (JMH) years of experience with cross-sectional imaging of the head and neck.

The area of the thyroid bed and cervical lymph node basins in the FDG-PET/ldCT and MRI examinations were analyzed for locally recurrent TC and nodal metastases separately as well as non-split. In order to be able to compare both imaging modalities, all pathology seen in the FDG-PET/ldCT, which was located outside the field of view covered by the MRI, was excluded for analysis. The test results were classified as either positive or negative based on a qualitative visual analysis. If either one or both of the two readers identified a pathologic finding, the result was considered to be positive. Uncertain findings were classified as negative. To explore the possibilities for a synergistic effect, the results of the FDG-PET/ldCT and MRI examinations were combined as follows: If either both FDG-PET/ldCT and MRI or one of each was positive, then the combined result was determined to be positive. If both the FDG-PET/ldCT and MRI findings were negative, then the combined result was classified as negative. The separate analyses were followed by a consensus reading between the FDG-PET/ldCT and MRI observers. Inter-observer agreement was determined by means of Cohen’s kappa coefficient (κ) and classified according to Landis and Koch [31].

#### Validity

The gold standard was defined as histopathological findings (subgroup a, *n* = 20) or, if the patient did not receive an operation for any reason, a negative long-term clinical follow-up period of at least 3 years (subgroup b, *n* = 26). In subgroup a 17 patients were operated as a result of the findings of the FDG-PET/ldCT and MRI examinations, whereas 3 patients underwent surgery in the subsequent clinical course. Due to the high number of patients who did not undergo surgery, we performed a separate subgroup analysis on this group. The gold standard for locally recurrent TC or nodal metastases was determined either as “positive” or “negative”, respectively.

#### Method comparisons for detection of locally recurrent TC and cervical nodal metastases

The findings of the FDG-PET/ldCT and MRI examinations were independently compared to the gold standard in a cross table analysis. Subsequently, the combined FDG-PET/ldCT and MRI results were compared to the gold standard. Finally, the results of the consensus reading were compared to the gold standard and to the combined FDG-PET/ldCT and MRI results. For each comparison, the sensitivity, specificity, positive predictive value (PPV), negative predictive value (NPV), and diagnostic accuracy were calculated.

#### Clinical relevance

To evaluate the clinical relevance of the detected imaging findings, we separately analyzed the therapeutic consequences of the PET/CT and MRI examinations in clinical routine. According to Wiebel et al. [32] the FDG-PET/ldCT and MRI scans were considered to impact the patient management if the “examinations identified disease that was not previously known and resulted in an intervention”, if the scans “resulted in the decision to not pursue a specific intervention”, or if the scan “changed the extent of a planned surgery because of additional disease identified” [32].

### Statistical tests

Data analyses were performed using IBM SPSS Statistics® Version 22 (IBM, NY, USA). Cohen’s kappa coefficient was used to determine inter-observer agreement. For method comparison, the sensitivity, specificity, PPV, and NPV were calculated, and McNemar’s test for paired samples was applied, where the level of significance was set at α = 0.05.

## Results

In the detection of both locally recurrent TC and nodal metastases with FDG-PET/ldCT, there was a substantial agreement between reader 1 and reader 2 (κ = 0.71 and κ = 0.63, respectively). In the non-split analysis of detection of locally recurrent TC or nodal metastases we found an almost perfect agreement between reader 1 and reader 2 (κ = 0.83).

In the detection of locally recurrent TC with MRI, there was a substantial agreement between reader 1 and reader 2 (κ = 0.69), whereas in the detection of nodal metastases with MRI there was only a moderate agreement (κ = 0.55). In the non-split analysis of detection of locally recurrent TC or nodal metastases we found a substantial agreement between reader 1 and reader 2 (κ = 0.67).

In the detection of both locally recurrent TC and nodal metastases, there was only slight to fair agreement between the findings from the FDG-PET/ldCT and MRI (κ = 0.21).

The sensitivity, specificity, PPV, NPV, and diagnostic accuracy for detecting locally recurrent TC or lymph node metastases are shown in Tables 2 and 3. The separate analyses for locally recurrent TC and nodal metastases are demonstrated in Additional files 1, 2, 3 and 4: Tables S1–S4.

**Table 2:** Detection of local recurrence or nodal metastases of thyroid cancer

Gold standard		FDG-PET/ldCT		MRI		Combined FDG-PET/ldCT and MRI		Consensus reading		
		-	+	-	+	-	+	-	+	Sum
HP	-	2	1	1	2	1	2	2	1	3
	+	2	15	7	10	1	16	1	16	17
	sum	4	16	8	12	2	18	3	17	20
FU	-	19	2	15	6	14	7	19	2	21
	+	0	5	3	2	0	5	0	5	5
	sum	19	7	18	8	14	12	19	7	26
Both	-	21	3	16	8	15	9	21	3	24
	+	2	20	10	12	1	21	1	21	22
	sum	23	23	26	20	16	30	22	24	46

Comparison of FDG-PET/ldCT, MRI, combined FDG-PET/ldCT and MRI, and consensus reading in detection of local recurrence or nodal metastases of thyroid cancer

Subgroup analysis of different gold standard

*HP* histopathology, *FU* follow-up

**Table 3:** Diagnostic performance of FDG-PET/ldCT, MRI, combined FDG-PET/ldCT and MRI, and the consensus reading

	FDG-PET/ldCT			MRI			combined FDG-PET/ldCT and MRI			consensus reading		
	HP	FU	Both	HP	FU	Both	HP	FU	Both	HP	FU	Both
Sensitivity	88 %	100 %	91 %	59 %	40 %	54 %	94 %	100 %	95 %	94 %	100 %	95 %
Specificity	67 %	90 %	87 %	33 %	71 %	67 %	33 %	67 %	62 %	67 %	90 %	87 %
PPV	94 %	71 %	87 %	83 %	25 %	60 %	89 %	42 %	70 %	94 %	71 %	87 %
NPV	50 %	100 %	91 %	12 %	83 %	61 %	50 %	100 %	94 %	67 %	100 %	95 %
Accuracy	85 %	92 %	89 %	55 %	65 %	61 %	85 %	73 %	78 %	90 %	92 %	91 %

Diagnostic performance of FDG-PET/ldCT, MRI, combined FDG-PET/ldCT and MRI, and the consensus reading in detection of local recurrence or nodal metastases of thyroid cancer

Subgroup analysis of different gold standard

*HP* histopathology, *FU* follow-up, *PPV* positive predictive value, *NPV* negative predictive value

The FDG-PET/ldCT and MRI results had impact on patient management in 23 of 46 patients (50 %): 14 patients (30 %) were operated on, 3 patients (7 %) underwent surgery and radiotherapy, 3 patients (7 %) were irradiated, 2 patients (4 %) underwent radio iodine therapy, and 1 patient (2 %) was treated with tyrosine kinase inhibitor therapy. In 10 patients (22 %) with active disease, an active surveillance strategy was chosen for various reasons, e.g., inoperable situation or poor general condition.

**Fig. 1:**
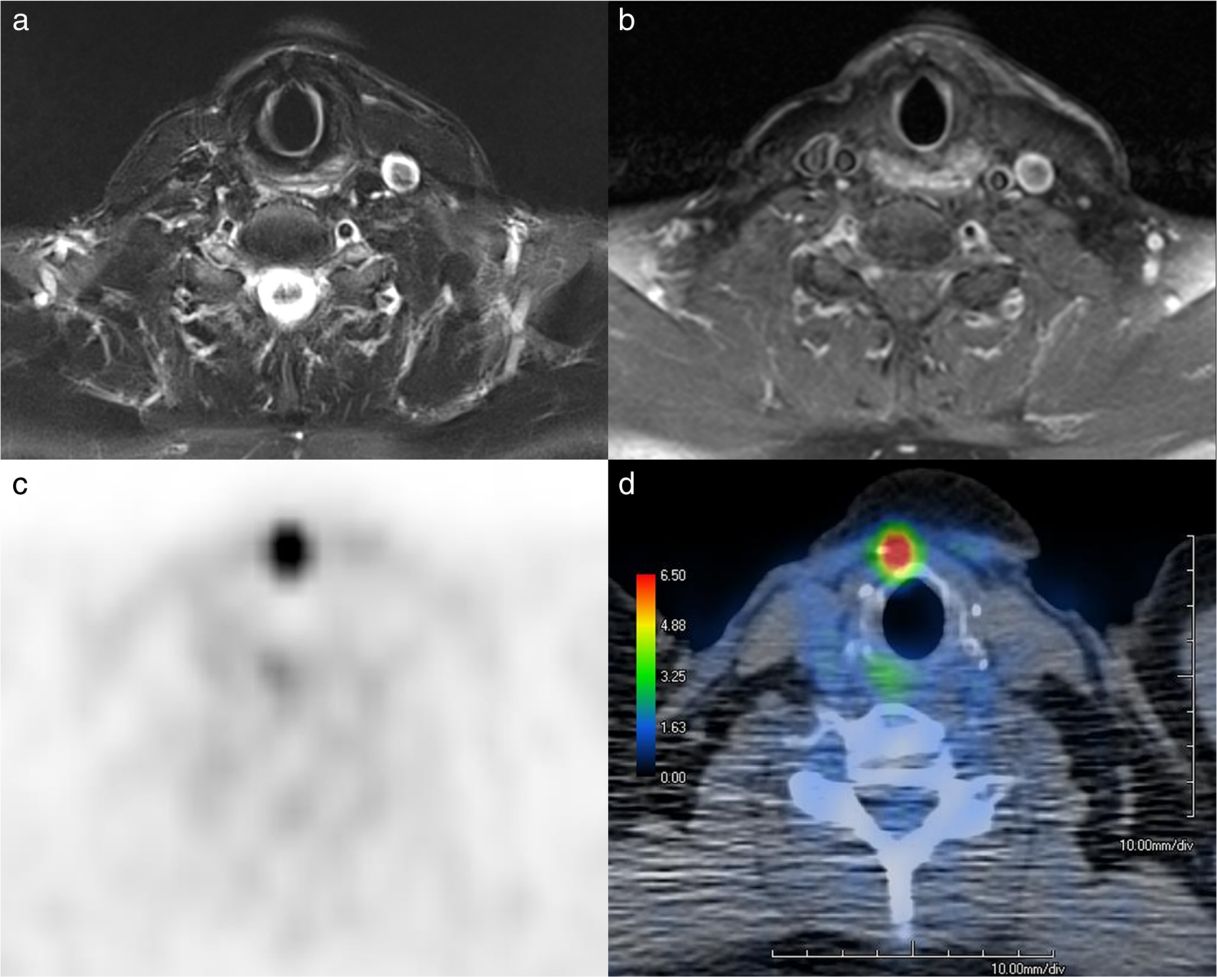
The added value of FDG-PET/ldCT for identifying a locally recurrent thyroid cancer with unspecific pretracheal finding via MRI. **a** STIR-weighted MR image, **b** contrast-enhanced T1-weighted MR image, **c** FDG-PET, and **d** fused FDG-PET and CT

Figure 1 shows the added value of FDG-PET/ldCT and MRI for clearer identification of a locally recurrent TC.

## Discussion

The purpose of this study was to investigate the impact of combined FDG-PET/ldCT and MRI on detecting locally recurrent TC and nodal metastases in high risk patients, with a special focus on the value of a multidisciplinary consensus reading.

The rise in use of diagnostic imaging, especially of FDG-PET(/CT) in TC follow-up, leads to increased radiation exposure and associated health costs [32–34]. Unnecessary diagnosis and intervention of low-risk and clinically non-significant disease remain a legitimate concern [34]. However, it is important to note that the imaging results analyzed in this study influenced patient management in over 50 % of patients, of whom 17 (37 %) were operated. The high percentage of high-risk patients with stage 4 disease may explain the higher percentage of changes in patient management than has been recently reported in the literature with approximately 30 % [32]. However, the smaller number of patients in our study may have caused selection bias.

Our data showed moderate inter-observer agreement in the detection of cervical nodal metastases with MRI. In our opinion, this moderate agreement is due to the fact that a reliable differentiation between malign and benign lymph nodes that is solely based on morphologic criteria is often not possible – a problem that is well-known in the literature [4, 35].

With regards to the data validity as compared to the heterogeneous gold standard there was a basic issue: If a recurrent disease was suspected, then the patient usually underwent surgery. Thus, over 80 % of the positive imaging results were confirmed by histopathology. In case of clearly negative FDG-PET/ldCT and MRI results, or if the patient was not suitable for surgery, no histologic sample could be gathered. As a consequence, the positive predictive value was higher and the negative predictive value was lower in the surgery subgroup.

Comparing the individual imaging modalities, FDG-PET/ldCT clearly outperformed MRI in detecting locally recurrent TC by having a higher sensitivity, specificity, PPV, NPV, and diagnostic accuracy. In the identification of cervical nodal metastases, FDG-PET/ldCT had a higher sensitivity and NPV than MRI, and an equal diagnostic accuracy. In non-split analysis FDG-PET/ldCT also clearly outperformed MRI by having a higher sensitivity, specificity, PPV, NPV, and diagnostic accuracy. Our overall FDG-PET/ldCT and MRI results were within the sensitivity and specificity range reported in the literature, where the metabolic PET component was described as “the leading tool for the detection of tumor recurrence, regardless of the anatomical imaging component with which it is combined” [26]. Nonetheless, the primary benefit of MRI lays in the precise morphologic assessment and correlation of metabolic foci, and its use in planning a surgical approach [7]. Additionally, MRI correctly identified locally recurrent TC in one patient with PET-negative disease.

The poor agreement between the FDG-PET/ldCT and MRI results (κ = 0.21) most likely originated from their basic methodic differences. In truth, both imaging modalities identified different true positive results and thus demonstrate a synergistic effect for combining these results. The combination of FDG-PET/ldCT and MRI test results primarily increased sensitivity and NPV. Nonetheless, specificity, PPV, and diagnostic accuracy decreased markedly due to concomitant higher rates of false positives. Thus, their combination is not appropriate for clinical routine, for it would increase the rate of subsequent interventions of potentially clinical non-relevant disease. We found that the multidisciplinary consensus reading was a key element in this dilemma. In mammography screenings, for instance, the value of second imaging and consensus reading has been extensively discussed in the literature, which finally led to its mandatory introduction in clinical practice [36, 37]. With regards to TC, the influence of consensus reading on the detection of locally recurrent tumor or nodal metastases has not yet been sufficiently investigated in the literature. Consensus interpretation in assessing FDG-PET/CT examinations have been reported to impact specificity by considerably reducing the number of false positive findings [11]. Correspondingly, for detecting local recurrence or nodal metastases of TC, the consensus reading outperformed the combined FDG-PET/ldCT and MRI results by increasing specificity, PPV, and diagnostic accuracy, while maintaining sensitivity and NPV. Specifically, 5 false positive MRI results with suspicious morphological findings as well as one false positive FDG-PET/ldCT result with FDG-negative disease could have been corrected by means of the consensus reading. However, it is of note that the consensus reading could improve the sensitivity, NPV, and diagnostic accuracy values of FDG-PET/ldCT only moderately, which in turn stresses the importance of metabolic PET imaging for detection of recurrent disease [26].

PET/MRI is a new emerging imaging modality with highly limited availability in clinical practice. In TC the first results suggest that FDG-PET/MRI is equal to contrast enhanced FDG-PET/CT in assessing cervical status. Nonetheless, FDG-PET/ldCT was shown to be superior to FDG-PET/MRI in assessing pulmonary status [30]. Therefore, Vrachimis et al. [30] suggested combined PET/MRI and low dose CT of the lung as a powerful diagnostic tool in patients with suspected recurrent TC. Our study results may support this approach, insofar as it combines the advantages of functional and morphological imaging with potentially highest possible level on diagnostic performance as well as least possible radiation dose. However, future studies should look deeper into the clinical benefit of PET/MRI in the follow-up care of post-thyroidectomy TC patients.

### Limitations

Our study patients were examined with different MRI scanners that had different field strengths, gradients, and examination protocols. Despite this limitation, there was a relatively high inter-observer agreement in the MRI group. Secondly, many MRI examinations were performed without additional diffusion-weighted imaging or functional imaging, which may have decreased the sensitivity and specificity of MRI. Thirdly, PET/CT was performed in a "whole-body" approach, while MRI was only performed in the neck region. This study focused on the neck area in order to be able to compare these two imaging modalities. Of course, a whole-body approach with PET/MRI allowing for a direct modality comparison with PET/CT would have been desirable, but was not feasible in our department. In addition, most of the selected patients were negative for active disease. Given the lower rate of positive findings, the sensitivity and specificity values were possibly subject to selection bias and the excellent sensitivity, specificity, and overall accuracy approaching 100 % may not hold up in larger samples. However, the number of patients with suspected recurrent or metastatic TC, negative US, and negative I-WBS is naturally limited. Nonetheless, the size of our sample (*n* = 46) is close to the calculated minimum sample size required for paired comparison (*n* = 54), indicating increased probability for Type I Error (α) of 0.05, a Power (1-β) of 0.9, and an expected sensitivity or specificity in group 1 of 0.95 and in group 2 of 0.8. Finally, the retrospective study design is unable to truly assess the impact of MRI and FDG-PET/ldCT on patient management, as clinical decisions were based upon the original reports of these scans and the review in multidisciplinary tumor board, not the retrospective consensus review.

## Conclusions

Our study results clearly demonstrate a synergistic effect for combining FDG-PET/ldCT and MRI in detecting locally recurrent TC and cervical nodal metastases in high-risk patients with negative ultrasound and I-WBS. Imaging results notably influenced patient management. Therefore, both complementary imaging modalities should be used together in this specific clinical setting. Finally, a multidisciplinary consensus reading between a nuclear medicine physician and a radiologist is a crucial element in this diagnostic approach.

## Additional files

Additional file 1: Table S1. Comparison of FDG-PET/CT, MRI, combined FDG-PET/CT and MRI, and consensus reading (locally recurrent thyroid cancer). Subgroup analysis of different gold standard; HP, histopathology; FU, follow-up; Separate cross table analysis for comparison of FDG-PET/CT, MRI, combined FDG-PET/CT and MRI, and consensus reading in detection of locally recurrent thyroid cancer. (DOCX 13 kb)
Additional file 2: Table S2. Diagnostic performance of FDG-PET/CT, MRI, combined FDG-PET/CT and MRI, and the consensus reading (local recurrence). Description: Subgroup analysis of different gold standard; HP, histopathology; FU, follow-up; PPV, positive predictive value; NPV, negative predictive value; Diagnostic performance of FDG-PET/ldCT, MRI, combined FDG-PET/ldCT and MRI, and consensus reading in separate analysis of detection of locally recurrent of thyroid cancer. (DOCX 13 kb)
Additional file 3: Table S3. Comparison of FDG-PET/CT, MRI, combined FDG-PET/CT and MRI, and consensus reading (cervical nodal metastases). Subgroup analysis of different gold standard; HP, histopathology; FU, follow-up; Separate cross table analysis for comparison of FDG-PET/CT, MRI, combined FDG-PET/CT and MRI, and consensus reading in detection of cervical nodal metastases. (DOCX 13 kb)
Additional file 4: Table S4. Diagnostic performance of FDG-PET/CT, MRI, combined FDG-PET/CT and MRI, and the consensus reading (nodal metastases). Subgroup analysis of different gold standard; HP, histopathology; FU, follow-up; PPV, positive predictive value; NPV, negative predictive value. Diagnostic performance of FDG-PET/ldCT, MRI, combined FDG-PET/ldCT and MRI, and consensus reading in separate analysis of detection of nodal metastases of thyroid cancer. (DOCX 13 kb)

## Abbreviations

FDG-PET: ^18^F-fluorodeoxyglucose Positron emission tomography
FDG-PET/CT: ^18^F-fluorodeoxyglucose-PET/computed tomography
FDG-PET/ldCT: ^18^F-fluorodeoxyglucose-PET/low dose computed tomography
FDG-PET/MRI: ^18^F-fluorodeoxyglucose-PET/MRI
FU: Follow-up
HP: Histopathology
I-WBS: ^131^I-whole-body scan
MRI: Magnetic resonance imagining
NPV: Negative predictive value
PPV: Positive predictive value
TC: Thyroid carcinoma
Tg: Thyroglobulin
US: Ultrasound

## References

[1] National Cancer Institute (U.S.). SEER Incidence Statistics - SEER Cancer Query Systems. 16.07.2015. http://seer.cancer.gov/canques/incidence.html. Accessed 27 Oct 2016.

[2] Rahib L, Smith BD, Aizenberg R, Rosenzweig AB, Fleshman JM, Matrisian LM (2014). Projecting Cancer Incidence and Deaths to 2030: The Unexpected Burden of Thyroid, Liver, and Pancreas Cancers in the United States. Cancer Res.

[3] Robert Koch Institut (2013). Beiträge zur Gesundheitsberichterstattung des Bundes - Krebs in Deutschland 2009/2010.

[4] Mihailovic J, Prvulovic M, Ivkovic M, Markoski B, Martinov D (2010). MRI versus ^131^I whole-body scintigraphy for the detection of lymph node recurrences in differentiated thyroid carcinoma. AJR Am J Roentgenol.

[5] Weber T, Ohlhauser D, Hillenbrand A, Henne-Bruns D, Reske SN, Luster M, Reske S (2012). Impact of FDG-PET computed tomography for surgery of recurrent or persistent differentiated thyroid carcinoma. Horm Metab Res.

[6] Song H-J, Xue Y-L, Xu Y-H, Qiu Z-L, Luo Q-Y (2011). Rare metastases of differentiated thyroid carcinoma: pictorial review. Endocr Relat Cancer.

[7] Haugen BR, Alexander EK, Bible KC, Doherty GM, Mandel SJ, Nikiforov YE (2016). 2015 American Thyroid Association Management Guidelines for Adult Patients with Thyroid Nodules and Differentiated Thyroid Cancer: The American Thyroid Association Guidelines Task Force on Thyroid Nodules and Differentiated Thyroid Cancer. Thyroid.

[8] Lind P, Kohlfurst S (2006). Respective roles of thyroglobulin, radioiodine imaging, and positron emission tomography in the assessment of thyroid cancer. Semin Nucl Med.

[9] Pacini F, Castagna MG, Brilli L, Pentheroudakis G, Group, on behalf of the ESMO Guidelines Working (2012). Thyroid cancer: ESMO Clinical Practice Guidelines for diagnosis, treatment and follow-up. Ann Oncol.

[10] Freudenberg LS, Antoch G, Frilling A, Jentzen W, Rosenbaum SJ, Kühl H (2008). Combined metabolic and morphologic imaging in thyroid carcinoma patients with elevated serum thyroglobulin and negative cervical ultrasonography: role of 124I-PET/CT and FDG-PET. Eur J Nucl Med Mol Imaging.

[11] Zoller M, Kohlfuerst S, Igerc I, Kresnik E, Gallowitsch H-J, Gomez I, Lind P (2007). Combined PET/CT in the follow-up of differentiated thyroid carcinoma: what is the impact of each modality?. Eur J Nucl Med Mol Imaging.

[12] Nahas Z, Goldenberg D, Fakhry C, Ewertz M, Zeiger M, Ladenson PW (2005). The role of positron emission tomography/computed tomography in the management of recurrent papillary thyroid carcinoma. Laryngoscope.

[13] Grünwald F, Kälicke T, Feine U, Lietzenmayer R, Scheidhauer K, Dietlein M (1999). Fluorine-18 fluorodeoxyglucose positron emission tomography in thyroid cancer: results of a multicentre study. Eur J Nucl Med.

[14] Dietlein M, Scheidhauer K, Voth E, Theissen P, Schicha H (1997). Fluorine-18 fluorodeoxyglucose positron emission tomography and iodine-131 whole-body scintigraphy in the follow-up of differentiated thyroid cancer. Eur J Nucl Med.

[15] Nagamachi S, Wakamatsu H, Kiyohara S, Nishii R, Mizutani Y, Fujita S (2011). Comparison of diagnostic and prognostic capabilities of (1)(8)F-FDG-PET/CT, (1)(3)(1)I-scintigraphy, and diffusion-weighted magnetic resonance imaging for postoperative thyroid cancer. Jpn J Radiol.

[16] Kim S-J, Lee TH, Kim I-J, Kim Y-K (2009). Clinical implication of F-18 FDG PET/CT for differentiated thyroid cancer in patients with negative diagnostic iodine-123 scan and elevated thyroglobulin. Eur J Radiol.

[17] Caetano R, Bastos CR, de Oliveira IA, da Silva RM, Fortes C, Pepe VL (2016). Accuracy of positron emission tomography and positron emission tomography-CT in the detection of differentiated thyroid cancer recurrence with negative (131) I whole-body scan results: A meta-analysis. Head Neck.

[18] Treglia G, Muoio B, Giovanella L, Salvatori M (2013). The role of positron emission tomography and positron emission tomography/computed tomography in thyroid tumours: an overview. Eur Arch Otorhinolaryngol.

[19] Nanni C, Rubello D, Fanti S, Farsad M, Ambrosini V, Rampin L (2006). Role of 18F-FDG-PET and PET/CT imaging in thyroid cancer. Biomed Pharmacother.

[20] Platzek I, Beuthien-Baumann B, Schneider M, Gudziol V, Langner J, Schramm G (2013). PET/MRI in head and neck cancer: initial experience. Eur J Nucl Med Mol Imaging.

[21] Bhargava P, Rahman S, Wendt J (2011). Atlas of Confounding Factors in Head and Neck PET/CT Imaging. Clin Nucl Med.

[22] Gross ND, Weissman JL, Talbot JM, Andersen PE, Wax MK, Cohen JI (2001). MRI detection of cervical metastasis from differentiated thyroid carcinoma. Laryngoscope.

[23] Nagarajah J, Jentzen W, Hartung V, Rosenbaum-Krumme S, Mikat C, Heusner TA (2011). Diagnosis and dosimetry in differentiated thyroid carcinoma using 124I PET: comparison of PET/MRI vs PET/CT of the neck. Eur J Nucl Med Mol Imaging.

[24] Dammann F, Bootz F, Cohnen M, Hassfeld S, Tatagiba M, Kösling S (2014). Diagnostic imaging modalities in head and neck disease. Dtsch Arztebl Int.

[25] Miyakoshi A, Dalley RW, Anzai Y (2007). Magnetic resonance imaging of thyroid cancer. Top Magn Reson Imaging.

[26] Queiroz MA, Hüllner M, Kuhn F, Huber G, Meerwein C, Kollias S (2014). PET/MRI and PET/CT in follow-up of head and neck cancer patients. Eur J Nucl Med Mol Imaging.

[27] Daftary A (2010). PET-MRI: Challenges and new directions. Indian J Nucl Med.

[28] Binse I, Poeppel TD, Ruhlmann M, Gomez B, Umutlu L, Bockisch A, Rosenbaum-Krumme SJ. Imaging with I in differentiated thyroid carcinoma: is PET/MRI superior to PET/CT? Eur J Nucl Med Mol Imaging. 2015. doi:10.1007/s00259-015-3288-y 10.1007/s00259-015-3288-y 26686334

[29] Dercle L, Deandreis D, Terroir M, Leboulleux S, Lumbroso J, Schlumberger M, et al. Evaluation of I PET/CT and I PET/MRI in the management of patients with differentiated thyroid cancer. Eur J Nucl Med Mol Imaging. 2016. doi:10.1007/s00259-016-3334-4 10.1007/s00259-016-3334-4 26928579

[30] Vrachimis A, Burg MC, Wenning C, Allkemper T, Weckesser M, Schafers M, Stegger L (2016). (18)FFDG PET/CT outperforms (18)FFDG PET/MRI in differentiated thyroid cancer. Eur J Nucl Med Mol Imaging.

[31] Landis JR, Koch GG (1977). The measurement of observer agreement for categorical data. Biometrics.

[32] Wiebel JL, Esfandiari NH, Papaleontiou M, Worden FP, Haymart MR, Wiebel JL, Worden FP (2015). Evaluating Positron Emission Tomography Use in Differentiated Thyroid Cancer. Thyroid.

[33] Gimbel RW, Fontelo P, Stephens MB, Olsen CH, Bunt C, Ledford CJW (2013). Radiation exposure and cost influence physician medical image decision making: a randomized controlled trial. Med Care.

[34] Wiebel JL, Banerjee M, Muenz DG, Worden FP, Haymart MR (2015). Trends in imaging after diagnosis of thyroid cancer. Cancer.

[35] Chen Q, Raghavan P, Mukherjee S, Jameson MJ, Patrie J, Xin W (2015). Accuracy of MRI for the diagnosis of metastatic cervical lymphadenopathy in patients with thyroid cancer. Radiol Med.

[36] Thurfjell EL, Lernevall KA, Taube AA (1994). Benefit of independent double reading in a population-based mammography screening program. Radiology.

[37] Anttinen I, Pamilo M, Soiva M, Roiha M (1993). Double reading of mammography screening films—one radiologist or two?. Clin Radiol.

