# Supplementary material for: Impact of combined FDG-PET/CT and MRI on the detection of local recurrence and nodal metastases in thyroid cancer
Source: Cancer Imaging. 2016 Nov 3;16:37. doi: 10.1186/s40644-016-0096-y (PMC5093960; doi:10.1186/s40644-016-0096-y)
Supplement: Additional file 1: Table S1. — Comparison of FDG-PET/CT, MRI, combined FDG-PET/CT and MRI, and consensus reading (locally recurrent thyroid cancer). Subgroup analysis of different gold standard; HP, histopathology; FU, follow-up; Separate cross table analysis for comparison of FDG-PET/CT, MRI, combined FDG-PET/CT and MRI, and consensus reading in detection of locally recurrent thyroid cancer. (DOCX 13 kb) [file 40644_2016_96_MOESM1_ESM.docx]

**Additional table 1** Comparison of FDG-PET/CT, MRI, combined FDG-PET/CT and MRI, and consensus reading (locally recurrent thyroid cancer)

| Gold standard | | FDG-PET/CT | | MRI | | Combined FDG-PET/CT and MRI | | Consensus reading | |  |
| --- | --- | --- | --- | --- | --- | --- | --- | --- | --- | --- |
|  |  | - | + | - | + | - | + | - | + | sum |
| HP | - | 5 | 2 | 6 | 1 | 5 | 2 | 5 | 2 | 7 |
|  | + | 4 | 9 | 7 | 6 | 2 | 11 | 0 | 13 | 13 |
|  | sum | 9 | 11 | 13 | 7 | 7 | 13 | 5 | 15 | 20 |
| FU | - | 22 | 1 | 18 | 5 | 18 | 5 | 21 | 2 | 23 |
|  | + | 1 | 2 | 2 | 1 | 0 | 3 | 0 | 3 | 3 |
|  | sum | 23 | 3 | 20 | 6 | 18 | 8 | 21 | 5 | 26 |
| Both | - | 27 | 3 | 24 | 6 | 23 | 7 | 26 | 4 | 30 |
|  | + | 5 | 11 | 9 | 7 | 2 | 14 | 0 | 16 | 16 |
|  | sum | 32 | 14 | 33 | 13 | 25 | 21 | 26 | 20 | 46 |

Subgroup analysis of different gold standard; HP, histopathology; FU, follow-up;

Separate cross table analysis for comparison of FDG-PET/CT, MRI, combined FDG-PET/CT and MRI, and consensus reading in detection of locally recurrent thyroid cancer.
