# Supplementary material for: Impact of combined FDG-PET/CT and MRI on the detection of local recurrence and nodal metastases in thyroid cancer
Source: Cancer Imaging. 2016 Nov 3;16:37. doi: 10.1186/s40644-016-0096-y (PMC5093960; doi:10.1186/s40644-016-0096-y)
Supplement: Additional file 2: Table S2. — Diagnostic performance of FDG-PET/CT, MRI, combined FDG-PET/CT and MRI, and the consensus reading (local recurrence). Description: Subgroup analysis of different gold standard; HP, histopathology; FU, follow-up; PPV, positive predictive value; NPV, negative predictive value; Diagnostic performance of FDG-PET/ldCT, MRI, combined FDG-PET/ldCT and MRI, and consensus reading in separate analysis of detection of locally recurrent of thyroid cancer. (DOCX 13 kb) [file 40644_2016_96_MOESM2_ESM.docx]

**Additional table 2** Diagnostic performance of FDG-PET/CT, MRI, combined FDG-PET/CT and MRI, and the consensus reading (local recurrence)

|  | FDG-PET/CT | | | MRI | | | combined FDG-PET/CT and MRI | | | consensus reading | | |
| --- | --- | --- | --- | --- | --- | --- | --- | --- | --- | --- | --- | --- |
|  | HP | FU | **Both** | HP | FU | **Both** | HP | FU | **Both** | HP | FU | **Both** |
| Sensitivity | 69% | 67% | **69%** | 46% | 33% | **44%** | 85% | 100% | **88%** | 100% | 100% | **100%** |
| Specificity | 71% | 96% | **90%** | 86% | 78% | **80%** | 71% | 78% | **77%** | 71% | 91% | **87%** |
| PPV | 82% | 67% | **79%** | 86% | 17% | **54%** | 85% | 37% | **67%** | 100% | 60% | **80%** |
| NPV | 56% | 96% | **84%** | 46% | 90% | **73%** | 71% | 100% | **92%** | 71% | 100% | **100%** |
| Accuracy | 70% | 92% | **83%** | 60% | 73% | **67%** | 80% | 81% | **80%** | 90% | 92% | **91%** |

Subgroup analysis of different gold standard; HP, histopathology; FU, follow-up;
PPV, positive predictive value; NPV, negative predictive value;

Diagnostic performance of FDG-PET/ldCT, MRI, combined FDG-PET/ldCT and MRI, and consensus reading in separate analysis of detection of locally recurrent of thyroid cancer.
