# Supplementary material for: Impact of combined FDG-PET/CT and MRI on the detection of local recurrence and nodal metastases in thyroid cancer
Source: Cancer Imaging. 2016 Nov 3;16:37. doi: 10.1186/s40644-016-0096-y (PMC5093960; doi:10.1186/s40644-016-0096-y)
Supplement: Additional file 3: Table S3. — Comparison of FDG-PET/CT, MRI, combined FDG-PET/CT and MRI, and consensus reading (cervical nodal metastases). Subgroup analysis of different gold standard; HP, histopathology; FU, follow-up; Separate cross table analysis for comparison of FDG-PET/CT, MRI, combined FDG-PET/CT and MRI, and consensus reading in detection of cervical nodal metastases. (DOCX 13 kb) [file 40644_2016_96_MOESM3_ESM.docx]

**Additional table 3** Comparison of FDG-PET/CT, MRI, combined FDG-PET/CT and MRI, and consensus reading (cervical nodal metastases)

| Gold standard | | FDG-PET/CT | | MRI | | Combined FDG-PET/CT and MRI | | Consensus reading | |  |
| --- | --- | --- | --- | --- | --- | --- | --- | --- | --- | --- |
|  |  | - | + | - | + | - | + | - | + | sum |
| HP | - | 5 | 5 | 7 | 3 | 3 | 7 | 8 | 2 | 9 |
|  | + | 1 | 9 | 5 | 5 | 1 | 9 | 2 | 8 | 11 |
|  | sum | 6 | 14 | 12 | 8 | 4 | 16 | 10 | 10 | 20 |
| FU | - | 20 | 3 | 20 | 3 | 17 | 6 | 23 | 0 | 23 |
|  | + | 0 | 3 | 1 | 2 | 0 | 3 | 0 | 3 | 3 |
|  | sum | 20 | 6 | 21 | 5 | 17 | 9 | 23 | 3 | 26 |
| Both | - | 25 | 8 | 27 | 6 | 20 | 13 | 31 | 2 | 32 |
|  | + | 1 | 12 | 6 | 7 | 1 | 12 | 2 | 11 | 14 |
|  | sum | 26 | 20 | 33 | 13 | 21 | 25 | 33 | 13 | 46 |

Subgroup analysis of different gold standard; HP, histopathology; FU, follow-up;

Separate cross table analysis for comparison of FDG-PET/CT, MRI, combined FDG-PET/CT and MRI, and consensus reading in detection of cervical nodal metastases.
