# Supplementary material for: Impact of combined FDG-PET/CT and MRI on the detection of local recurrence and nodal metastases in thyroid cancer
Source: Cancer Imaging. 2016 Nov 3;16:37. doi: 10.1186/s40644-016-0096-y (PMC5093960; doi:10.1186/s40644-016-0096-y)
Supplement: Additional file 4: Table S4. — Diagnostic performance of FDG-PET/CT, MRI, combined FDG-PET/CT and MRI, and the consensus reading (nodal metastases). Subgroup analysis of different gold standard; HP, histopathology; FU, follow-up; PPV, positive predictive value; NPV, negative predictive value. Diagnostic performance of FDG-PET/ldCT, MRI, combined FDG-PET/ldCT and MRI, and consensus reading in separate analysis of detection of nodal metastases of thyroid cancer. (DOCX 13 kb) [file 40644_2016_96_MOESM4_ESM.docx]

**Additional table 4** Diagnostic performance of FDG-PET/CT, MRI, combined FDG-PET/CT and MRI, and the consensus reading (nodal metastases)

|  | FDG-PET/CT | | | MRI | | | combined FDG-PET/CT and MRI | | | consensus reading | | |
| --- | --- | --- | --- | --- | --- | --- | --- | --- | --- | --- | --- | --- |
|  | Sur | FU | **Both** | Sur | FU | **Both** | Sur | FU | **Both** | Sur | FU | **Both** |
| Sensitivity | 90% | 100% | **92%** | 50% | 67% | **54%** | 90% | 100% | **92%** | 73% | 100% | **85%** |
| Specificity | 50% | 87% | **76%** | 70% | 87% | **82%** | 30% | 74% | **61%** | 78% | 100% | **94%** |
| PPV | 65% | 50% | **60%** | 62% | 40% | **54%** | 56% | 33% | **48%** | 80% | 100% | **85%** |
| NPV | 83% | 100% | **96%** | 58% | 95% | **82%** | 75% | 100% | **95%** | 70% | 100% | **94%** |
| Accuracy | 70% | 88% | **80%** | 65% | 85% | **74%** | 60% | 77% | **70%** | 75% | 100% | **91%** |

Subgroup analysis of different gold standard; HP, histopathology; FU, follow-up;
PPV, positive predictive value; NPV, negative predictive value

Diagnostic performance of FDG-PET/ldCT, MRI, combined FDG-PET/ldCT and MRI, and consensus reading in separate analysis of detection of nodal metastases of thyroid cancer.
